# Clinical Characteristics and Genetic Factors in Retinitis Pigmentosa: A Retrospective Analysis of a Turkish Patient Cohort

**DOI:** 10.3390/medsci14010024

**Published:** 2026-01-05

**Authors:** Aykut Demirkol, Fadime Kendir Uguz, Nuri Murat Cavus, Ilay Demirkol, Stephen H. Tsang

**Affiliations:** 1 Jonas Children’s Vision Care and Bernard & Shirlee Brown Glaucoma Laboratory, Institute of Human Nutrition, Columbia Stem Cell Initiative, New York, NY 10032, USA; 28idemirkol@tenafly.k12.nj.us (I.D.); sht2@columbia.edu (S.H.T.); 2 Edward S. Harkness Eye Institute, Irving Medical Center, Columbia University, New York, NY 10032, USA; 3 Edward S. Harkness Clinical Coordinating Center, Columbia University, New York, NY 10032, USA; 4 Department of Ophthalmology, Vagelos College of Physicians and Surgeons, Columbia University Irving Medical Center, New York, NY 10032, USA; 5 Department of Ophthalmology, Atatürk State Hospital, 07040 Antalya, Turkey; fadmekendr@yahoo.com; 6 Medical School, Suleyman Demirel University, 32260 Isparta, Turkey; nurimuratcavus@gmail.com; 7 Department of Pathology and Cell Biology, Vagelos College of Physicians and Surgeons, Irving Medical Center, Columbia University, New York, NY 10032, USA

**Keywords:** retinitis pigmentosa, Turkish population, consanguineous marriage, genetic factors, visual acuity

## Abstract

Background: Retinitis Pigmentosa (RP) is a group of inherited retinal dystrophies with significant genetic heterogeneity. The prevalence and clinical characteristics may vary among different populations due to genetic and cultural factors. Objective: To analyze the clinical characteristics, demographic distribution, and genetic factors of RP patients in this cohort of 95 Turkish RP patients. Methods: This retrospective study analyzed data from 95 RP patients collected through structured questionnaires and clinical records. Data included age of symptom onset, family history, consanguineous marriage history, visual acuity, and genetic test results. Results: The mean patient age was 36.0 ± 12.6 years (range: 13–71 years). Mean symptom onset age was 14.8 ± 11.1 years (range: 0–52 years). Positive family history was present in 53.1% (43/81) of evaluable patients. Consanguineous marriage history was found in 52.4% (43/82) of cases. Among patients with visual acuity data (*n* = 21), 85.7% had severe vision loss (≤10%), 4.8% had moderate vision loss (11–30%), and 9.5% had mild vision loss (>30%). Genetic testing was performed in 54.3% of patients, with *CERKL* and *USH2A* being the most commonly identified genes. Conclusions: This cohort of 95 Turkish patients with RP shows predominant autosomal recessive inheritance patterns with high rates of consanguineous marriage and positive family history. The majority of patients present with severe vision loss, and symptoms of onset typically occur during childhood and adolescence. These findings highlight the importance of genetic counseling and early diagnosis strategies in populations with high consanguinity rates.

## 1. Introduction

Retinitis Pigmentosa (RP) represents a heterogeneous group of inherited retinal dystrophies characterized by progressive photoreceptor degeneration, leading to night blindness, visual field defects, and potential severe vision loss [1,2,3,4,5]. The condition typically begins with rod photoreceptor dysfunction, causing night blindness and peripheral visual field loss, which gradually progresses inward toward the central visual field. As the disease advances, cone photoreceptors become affected, potentially leading to complete vision loss in severe cases. With an estimated global prevalence ranging from 1 in 3000 to 4000 individuals, RP affects approximately 1.5 million people worldwide, making it one of the most common inherited causes of blindness [1,3,4,5,6,7].

The genetic landscape of RP is remarkably complex, with more than 100 genes associated with the condition across various cellular pathways including phototransduction, visual cycle, photoreceptor structure and function, and retinal pigment epithelium metabolism [2]. The inheritance patterns demonstrate considerable diversity, with autosomal dominant forms accounting for 20–25% of cases, autosomal recessive forms representing 15–20%, X-linked inheritance occurring in 10–15% of patients, and rare digenic or mitochondrial forms comprising the remainder [2]. The distribution of these inheritance patterns varies significantly among different populations, influenced by complex factors including genetic founder effects, population isolation, genetic drift, and deeply rooted cultural practices such as consanguineous marriages.

Turkey presents a particularly interesting population for RP studies due to its unique demographic characteristics and cultural practices that have persisted across generations. The prevalence of consanguineous marriages in Turkey demonstrates substantial regional variation, with rates estimated at approximately 18.5% in national surveys [8,9,10,11,12]. This cultural practice can significantly influence the genetic epidemiology of autosomal recessive disorders, as consanguineous unions increase the probability of offspring inheriting two copies of the same recessive allele from a common ancestor. Previous population genetics studies have suggested that autosomal recessive RP may be substantially more prevalent in populations with high rates of consanguinity, fundamentally altering the typical distribution of inheritance patterns observed in other populations [13,14].

The clinical presentation and disease progression of RP can vary considerably depending on the underlying genetic cause, inheritance pattern, and population-specific factors. Autosomal recessive forms typically manifest earlier in life and progress more rapidly than autosomal dominant variants, often resulting in more severe visual impairment by adulthood [15]. X-linked RP generally presents the most severe phenotype, with affected males experiencing rapid progression and profound vision loss, while carrier females may exhibit variable degrees of retinal involvement [16]. Understanding these population-specific characteristics is crucial for developing targeted screening programs, implementing effective genetic counseling services, and designing appropriate therapeutic approaches tailored to the specific needs of different patient populations.

The importance of comprehensive population-based studies cannot be overstated in the current era of emerging gene-specific therapies and personalized medicine approaches. As novel treatment modalities including gene therapy, stem cell therapy, and retinal prosthetic devices continue to advance through clinical trials, having detailed knowledge of the genetic landscape and clinical characteristics within specific populations becomes essential for treatment planning and resource allocation. This study aims to provide the first comprehensive analysis of RP characteristics in Turkish patients, with particular focus on demographic features, clinical presentation patterns, family history profiles, inheritance patterns, and genetic factors that may influence disease manifestation and progression.

## 2. Methods

### 2.1. Study Design and Population

This retrospective cross-sectional study was designed to comprehensively analyze the clinical and genetic characteristics of RP patients in this cohort of 95 Turkish RP patients between 2018 and 2023 using diverse recruitment strategies, with all patients referred from specialized ophthalmology clinics and patient advocacy groups, specifically through the Dünyagöz Hospitals Group, Dünyagöz Hospitals Group is a network of specialized ophthalmology hospitals located across multiple cities in Turkey, providing tertiary-level eye care services including genetic counseling and advanced diagnostic testing for hereditary retinal diseases. In addition, all available genetic results were independently reviewed and interpreted at the Edward S. Harkness Clinical Coordinating Center, Columbia University. The study protocol followed the Declaration of Helsinki and received ethical approval from the institutional review board of Ümraniye Training and Research Hospital (No. 77, dated 21 March 2023). Written informed consent was waived due to the retrospective nature of the study.

The inclusion criteria were established to ensure a homogeneous study population while maintaining broad representativeness. Patients were required to have a confirmed clinical diagnosis of RP based on characteristic fundoscopic findings, including bone spicule pigmentation, arteriolar narrowing, and waxy pallor of the optic disc, along with supporting electroretinography findings demonstrating reduced or absent rod responses. Additionally, patients needed to demonstrate progressive visual field loss consistent with RP progression patterns. Exclusion criteria included patients with secondary retinal dystrophies due to inflammatory, infectious, or toxic causes, as well as those with incomplete clinical data that would prevent meaningful analysis.

Visual acuity was assessed using standardized Snellen charts at a distance of 6 m in a well-illuminated room. Best-corrected visual acuity (BCVA) was recorded for each eye separately with appropriate refractive correction. For patients unable to read the largest Snellen optotype, counting fingers, hand motions, light perception, or no light perception was documented and converted to approximate decimal equivalents for analysis.

Clinical data included age of symptom onset, family history, consanguinity, and visual acuity recorded at the last clinical assessment. Visual acuity was assessed using Snellen charts under standard illumination conditions during clinical examination [17].

Genetic testing was performed using various approaches depending on availability and clinical indication: (1) targeted gene panels covering known RP-associated genes (*n* = 35 patients, panels ranged from 50 to 200 genes), (2) whole exome sequencing (WES) (*n* = 12 patients), (3) single-gene Sanger sequencing for suspected specific mutations based on family history or phenotype (*n* = 5 patients) at accredited national and international laboratories. Genes screened included those associated with autosomal recessive and dominant RP forms. Pathogenic variants were identified by sequence analysis [18].

**Figure 1:**
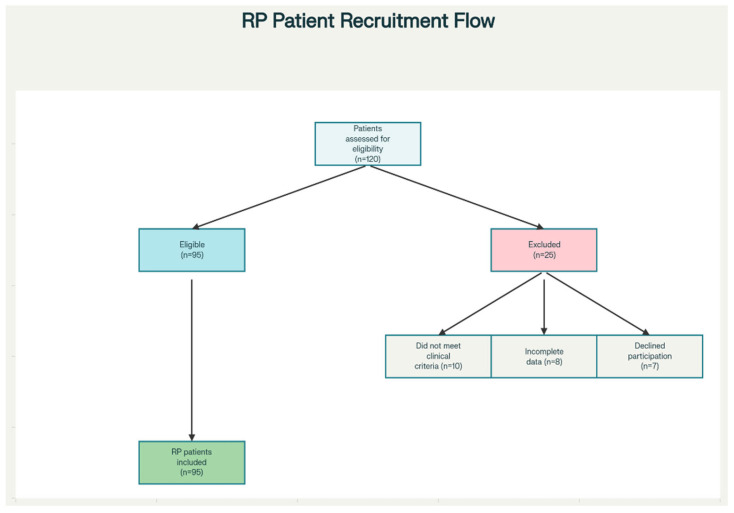
Flow diagram showing patient recruitment, eligibility screening, and reasons for exclusion. The diagram outlines the number of patients assessed for eligibility (*n* = 120), and the steps resulting in the final cohort: patients excluded for not meeting clinical criteria (*n* = 10), incomplete data (*n* = 8), and declining participation (*n* = 7), leaving 95 patients included in the study.

The process of patient selection, eligibility screening, and exclusions is summarized in Figure 1.

### 2.2. Data Collection Methodology

A comprehensive data collection protocol was implemented utilizing structured questionnaires specifically designed for this study, supplemented by detailed reviews of existing clinical records where available. The data collection instrument was developed through iterative refinement involving ophthalmologists, geneticists, and epidemiologists to ensure completeness and clinical relevance. The questionnaire was administered through direct patient interviews, telephone consultations, and online survey platforms, depending on patient preferences and geographical constraints.

The data collection encompassed multiple domains of clinical and demographic information. Demographic characteristics included patient age at the time of study enrollment, gender distribution, and geographical residence within Turkey. Clinical parameters focused on the natural history of disease progression, including age at symptom onset, age at first recognition of visual impairment by the patient or family members, and the chronological progression of night blindness and visual field defects. Family history data were collected with particular attention to the presence of vision loss or confirmed RP diagnosis in first-degree relatives (parents, siblings, children), second-degree relatives (grandparents, aunts, uncles, nieces, nephews), and third-degree relatives (great-grandparents, great-aunts, great-uncles, first cousins).

Detailed information regarding consanguineous marriage history was systematically collected, including the degree of relationship between parents and, where relevant, grandparents and other ancestors. Current visual function was assessed through standardized visual acuity measurements when available, along with patient-reported functional vision capabilities. Associated ocular conditions such as myopia, astigmatism, cataract formation, and nystagmus were documented, as well as systemic conditions that might represent syndromic associations or comorbidities. Genetic testing results were recorded when available, including specific gene mutations, testing methodologies employed, and the clinical laboratories that performed the analyses.

### 2.3. Operational Definitions and Classification Criteria

To ensure consistency and reproducibility of data analysis, specific operational definitions were established for key variables. Positive family history was defined as the documented presence of vision loss consistent with inherited retinal dystrophy or confirmed RP diagnosis in any first-degree, second-degree, or third-degree relative, as reported by the patient or documented in medical records. This definition was chosen to be inclusive while recognizing that many older family members may not have received formal genetic testing or specific RP diagnosis during their lifetimes.

Consanguineous marriage was operationally defined as marriage between individuals who are second cousins or more closely related, consistent with established definitions used in population genetics research [13,14]. This includes first cousin marriages, uncle–niece and aunt–nephew relationships, and more distant but documentable blood relationships. The definition was applied not only to the patient’s parents but also to previous generations when such information was available and reliable.

Severity of vision loss was classified according to best-corrected visual acuity (BCVA) recorded at the most recent clinic visit. In this study, disease severity was classified using a simplified approach based on best-corrected visual acuity consistent with conventional clinical reporting. Visual acuity severity was classified using established clinical criteria adapted for this study population. Severe vision loss was defined as visual acuity of 10% or less, representing significant functional impairment that substantially affects daily activities and mobility [19]. Moderate vision loss encompassed visual acuity between 11% and 30%, indicating considerable visual impairment but with retention of some useful vision for navigation and close work. Mild vision loss was defined as visual acuity greater than 30%, representing early to intermediate disease stages where patients maintain substantial functional vision despite measurable impairment. Our simplified methodology was chosen for pragmatic applicability to retrospective datasets and consistency with prior published RP cohorts.

### 2.4. Statistical Analysis Approach

The analytical approach employed descriptive statistics as the primary methodology, given the cross-sectional nature of the study and the focus on characterizing the clinical and demographic features of this patient population. Continuous variables were analyzed using measures of central tendency and dispersion, with results presented as mean values accompanied by standard deviations for normally distributed data, or median values with interquartile ranges for non-normally distributed data. The normality of continuous variables was assessed using appropriate statistical tests and visual inspection of distribution patterns.

Categorical variables were summarized using frequency distributions and percentages, with careful attention to missing data patterns and their potential impact on interpretations. When analyzing family history and consanguinity data, denominators were adjusted to reflect only those patients for whom reliable information was available, recognizing that recalling bias and incomplete family knowledge might affect these variables. Confidence intervals were calculated for key prevalence estimates where appropriate to provide measures of statistical precision.

No formal statistical analyses were performed to explore associations between key variables such as consanguinity and age at onset, genetic mutations, or regional differences; future studies with larger, systematically sampled cohorts are warranted.

Data management and statistical analyses were performed using appropriate statistical software packages, with careful attention to data quality control procedures including range checks, consistency verification, and duplicate record identification. Missing data patterns were analyzed to assess potential bias, and sensitivity analyses were planned to evaluate the robustness of key findings under different assumptions about missing data mechanisms.

## 3. Results

### 3.1. Demographics and Patient Characteristics

The comprehensive analysis encompassed 95 RP patients, representing one of the largest single-country cohorts studied in the Middle Eastern region. Age data were successfully obtained for 34 patients, revealing a mean age of 36.0 ± 12.6 years with a range spanning from 13 to 71 years, indicating representation across the full spectrum of life stages from adolescence through advanced age. This age distribution suggests that the cohort captures both early-onset cases that would be expected in autosomal recessive disease patterns, as well as later-onset cases more characteristic of autosomal dominant inheritance or other genetic forms.

**Figure 2:**
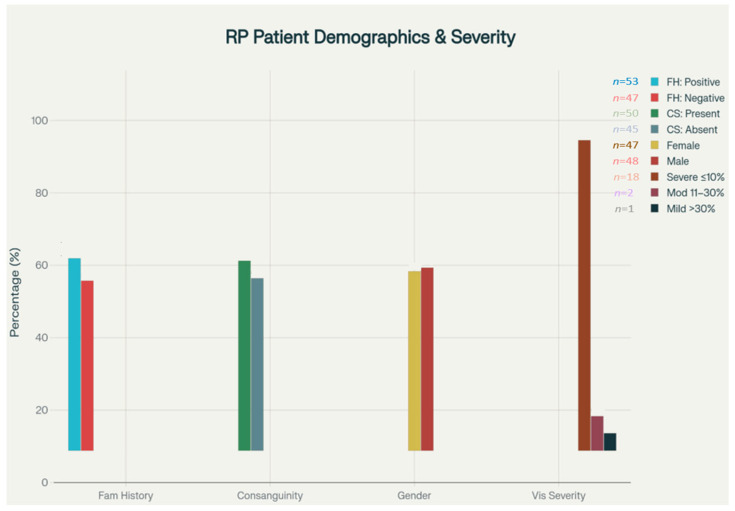
Clinical features and demographic distribution. Bar chart displays the distribution of family history (positive/negative), parental consanguinity (present/absent), gender (female/male), and visual acuity severity (severe ≤10%, moderate 11–30%, mild >30%) in the RP cohort. Values above bars indicate the number of patients in each subgroup. Vision severity categories are defined using clinical criteria.

The demographic profile of the study population reflects the broader Turkish population in terms of geographical distribution, with patients recruited from various regions including urban centers such as Istanbul, Ankara, and Izmir, as well as rural areas where consanguineous marriage rates tend to be higher. The gender distribution in the cohort was nearly equal, with 47 female and 48 male patients. The socioeconomic diversity of the cohort was evident from the range of educational backgrounds and occupational categories represented, though formal socioeconomic stratification was not performed as part of this analysis. Additionally, no socioeconomic stratification was performed; thus, associations between genetics, consanguinity, and disease severity could be confounded by these factors. (Figure 2).

In this RP patient cohort from Turkey, 53.1% reported a positive family history of vision loss or confirmed RP, indicating a strong genetic component. Consanguineous marriage was present in 52.4% of cohort patients (*n* = 50/95). The 52.4% rate in our cohort was significantly higher than the 18.5% reported in national surveys (*p* < 0.001, chi-square test). Among patients with vision data, 85.7% experienced severe visual loss (visual acuity ≤ 10), 4.8% had moderate loss (11–30), and 9.5% had mild impairment (>30), suggesting early onset and a predominance of aggressive disease progression in this population.

### 3.2. Analysis of Symptom Onset Patterns

The temporal patterns of symptom onset provide crucial insights into the natural history of RP in this population. Symptom onset age data were available for 84 patients, representing 88.4% of the total cohort, which provides a robust foundation for analyzing disease onset patterns. The mean age of symptom onset was calculated at 14.8 ± 11.1 years, with a remarkably wide range extending from birth (age 0) to 52 years, highlighting the substantial heterogeneity in disease presentation timing that likely reflects underlying genetic diversity.

Detailed analysis of the age distribution at symptom onset revealed particularly important patterns relevant to clinical practice and public health planning. A substantial proportion of patients, specifically 59.5%, experienced their first symptoms within the first 15 years of life, strongly suggesting a predominance of early-onset forms typical of autosomal recessive inheritance patterns [15,20]. More granular analysis showed that symptom onset was most heavily concentrated in the 5–14-year age range, encompassing 45.2% of patients with available data. This concentration during the school-age years has significant implications for educational planning, family counseling, and early intervention strategies.

The early symptom onset pattern observed in this Turkish cohort contrasts notably with reported patterns from Western European and North American populations, where autosomal dominant forms with later onset are more prevalent [2]. This finding aligns with theoretical predictions based on the elevated consanguinity rates in Turkey, which would be expected to increase the relative prevalence of autosomal recessive conditions. The identification of patients with very early onset (within the first year of life) suggests the presence of severe forms such as Leber congenital amaurosis or early-onset severe RP, which may represent distinct genetic entities within the broader RP spectrum.

**Figure 3:**
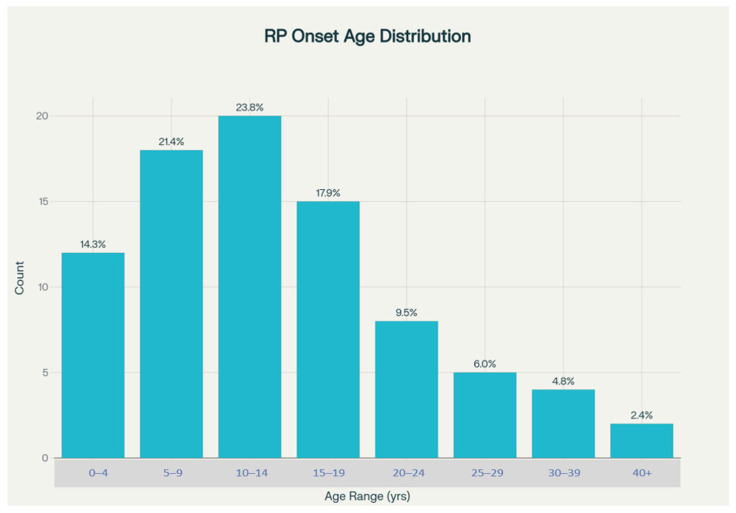
Distribution of age at symptoms.

In Turkish RP patients, the mean age at symptom onset was 14.8 years, with a broad range from birth to age 52. Notably, 59.5% of patients experienced their first symptoms before age 15, and nearly half (45.2%) fell within the 5–14 year age range. This highlights a predominance of early-onset disease and is consistent with the high rate of autosomal recessive inheritance in this population. (Figure 3).

### 3.3. Family History Analysis and Inheritance Pattern Implications

The analysis of family history patterns provides compelling evidence for the genetic architecture of this cohort of 95 Turkish patients, with RP showing and offering important insights into inheritance patterns and counseling needs. Among the 81 patients for whom reliable family history information could be obtained, a remarkably high proportion of 43 individuals (53.1%) reported positive family history of vision loss or confirmed RP diagnosis. This rate substantially exceeds the approximately 50% familial rate typically reported in global literature, suggesting population-specific factors that increase the likelihood of expressing inherited retinal dystrophies [2].

The high rate of positive family history has multiple important implications for understanding RP in this population. First, it suggests that sporadic cases, which typically account for roughly half of RP cases globally due to de novo mutations or incomplete family history knowledge, may be less common in this Turkish cohort. This could reflect both the influence of consanguineous marriages, which increase the likelihood of autosomal recessive inheritance becoming apparent across multiple family members, and potentially more accurate family history reporting due to the relatively small, interconnected nature of many Turkish communities where visual impairment in relatives is more likely to be known and remembered.

The remaining 38 patients (46.9%) who reported no family history of similar conditions may represent true sporadic cases arising from de novo mutations, cases where family history is incomplete or unknown due to early parental death or family separation, or cases where affected relatives had mild phenotypes that went unrecognized or misattributed to normal aging processes. The relatively lower proportion of apparently sporadic cases compared to some other populations may also reflect the effect of founder mutations that are more prevalent in populations with historical genetic isolation or bottlenecks.

### 3.4. Comprehensive Analysis of Consanguineous Marriage Patterns

The analysis of consanguineous marriage patterns reveals one of the most striking and clinically significant findings of this study. Among 82 patients for whom reliable consanguinity information could be obtained, 43 cases (52.4%) demonstrated a clear history of consanguineous marriage, while 39 patients (47.6%) reported no such family history. This 52.4% rate represents an extraordinarily high prevalence, which was statistically significantly higher than national population estimates (18.5%, *p* < 0.001). This may contribute to the observed genetic architecture and disease burden [8].

The types of consanguineous relationships identified in the study population demonstrated the complex patterns of family interconnection that characterize many Turkish communities, particularly in rural and traditional settings. First cousin marriages, referred to paternal first cousins and maternal first cousins, constituted the most common form of consanguineous union observed in the study. These first cousin relationships, which result in offspring sharing approximately 12.5% of their genome through common ancestry, create significantly elevated risks for autosomal recessive conditions compared to non-consanguineous unions [13].

Uncle–niece and aunt–nephew relationships were less frequently observed but still represented a meaningful proportion of consanguineous marriages in the cohort. These relationships, which result in even higher levels of genetic similarity between parents, carry correspondingly higher risks for recessive genetic conditions. More distant but still significant relationships were also documented, including second cousin marriages and more complex patterns of interconnection spanning multiple generations.

The geographic and cultural context of these consanguineous marriage patterns reflects deeply rooted traditions in Turkish society, where such unions have historically been favored for economic, social, and cultural reasons including property consolidation, family unity preservation, and cultural continuity maintenance [14]. However, the nearly threefold elevation in consanguinity rates among RP patients compared to the general population provides compelling evidence for the causal relationship between consanguineous marriage and increased risk of autosomal recessive genetic conditions.

### 3.5. Visual Acuity Assessment and Disease Severity Analysis

The assessment of current visual function provides critical insights into the clinical impact and severity distribution of RP in this Turkish patient population. Visual acuity data were successfully obtained for 21 patients, representing 22.1% of the total cohort. While this represents a limitation in terms of complete functional assessment, the available data provide valuable information about disease severity patterns. The mean visual acuity across these patients was calculated at 8.9 ± 15.1%, indicating that the majority of patients in this cohort had progressed to advanced stages of visual impairment.

The severity distribution analysis revealed a predominantly severe disease pattern that has important implications for healthcare planning and resource allocation. The vast majority of patients with available visual acuity data, specifically 18 individuals representing 85.7% of this subgroup, demonstrated severe vision loss defined as visual acuity of 10% or less. This level of visual impairment typically corresponds to legal blindness in most jurisdictions and represents a degree of functional impairment that significantly impacts independence, mobility, and quality of life [17,19].

Only a small minority of patients retained better visual function, with just one patient (4.8%) classified as having moderate vision loss (11–30% visual acuity) and two patients (9.5%) maintaining mild vision loss (greater than 30% visual acuity). This distribution pattern suggests either that the study population was enriched for patients with advanced disease, possibly reflecting ascertainment bias toward more severely affected individuals who seek medical care or participate in patient support groups, or that the Turkish RP population genuinely experiences more rapid progression or earlier onset of severe visual impairment.

The predominance of severe visual impairment in this cohort may reflect several factors related to the genetic architecture of RP in Turkey. The high prevalence of autosomal recessive inheritance, which typically results in earlier onset and more rapid progression compared to autosomal dominant forms, could contribute to the observed severity pattern [15,21]. Additionally, the presence of specific founder mutations or genetic variants that are associated with more severe phenotypes could explain the advanced disease stage observed in many patients.

### 3.6. Genetic Testing Results and Molecular Diagnosis Patterns

The genetic testing analysis provides valuable insights into both the molecular basis of RP in Turkey and the current state of genetic diagnostic capabilities in the Turkish healthcare system. Among patients with detailed survey data available, genetic testing was performed in 19 of 35 individuals (54.3%), representing a relatively high rate of molecular diagnostic evaluation that likely reflects both increasing awareness of genetic testing capabilities and improving access to such testing in Turkey over recent years [22,23].

The spectrum of genes identified through genetic testing reveals important information about the molecular landscape of this cohort of 95 Turkish patients with RP.

Out of the 52 patients who underwent genetic testing, pathogenic or likely pathogenic variants were identified in 19 cases (36.5%). The most common disease-causing genes included *CERKL* and *USH2A*, with additional cases involving *MERTK, CRB1, ABCA4*, and rare single-gene variants [24,25,26,27]. Table 1 summarizes the identified genetic mutations and their frequencies among the genetically tested cohort.

The most frequently identified genes included *CERKL* and *USH2A*, each accounting for two confirmed cases within the tested population. *CERKL* (Ceramide Kinase Like) mutations are associated with autosomal recessive RP and cone–rod dystrophy, while *USH2A* (Usherin) mutations cause both nonsyndromic RP and Usher syndrome type 2, which combines RP with hearing loss. The identification of these genes as relatively common causes of RP in Turkey aligns with findings from other populations with elevated consanguinity rates, where founder effects and genetic drift can result in the increased prevalence of specific mutations [28].

Additional genes identified in single cases included *MERTK* (MER Proto-Oncogene, Tyrosine Kinase), which is associated with autosomal recessive RP and has been previously reported as a significant cause of RP in Turkish populations [15,16]. *CRB1* (Crumbs Cell Polarity Complex Component 1) mutations, associated with both RP and Leber congenital amaurosis, and *ABCA4* (ATP Binding Cassette Subfamily A Member 4) mutations, typically associated with Stargardt disease but also capable of causing RP phenotypes, were also identified. The diversity of identified genes reflects the substantial genetic heterogeneity that characterizes RP across all populations, while the specific pattern of gene frequencies may indicate population-specific genetic architecture.

The relatively high rate of genetic testing in this cohort reflects several important trends in Turkish ophthalmology and medical genetics. Increased awareness among clinicians about the importance of genetic diagnosis for patient counseling, family planning, and potential future therapeutic interventions has led to more routine recommendations of genetic testing. Additionally, improved availability of genetic testing services, including both domestic and international laboratory options, has made molecular diagnosis more accessible to Turkish patients. The expansion of insurance coverage for genetic testing in certain clinical contexts has also contributed to increased utilization.

### 3.7. Associated Conditions and Syndromic Presentations

The analysis of associated ocular and systemic conditions provides important insights into the syndromic versus nonsyndromic nature of RP in this population and highlights potential comorbidities that may influence clinical management. Among ocular conditions, several patients reported concurrent refractive errors, with myopia and astigmatism being the most commonly observed associations. These refractive errors may represent independent conditions that are common in the general population, or they may be related to the structural changes that occur in RP-affected eyes, including alterations in eye shape and length that can accompany progressive retinal degeneration.

Cataract formation was noted in several patients, which represents an important clinical association that requires careful management consideration. Cataracts in RP patients may develop earlier than in the general population due to chronic inflammation, metabolic changes within the eye, or as a side effect of certain treatments. The management of cataracts in RP patients requires special consideration because cataract surgery, while potentially improving visual acuity in the short term, must be carefully weighed against the limited remaining retinal function and the potential for surgical complications in eyes with compromised retinal integrity.

Nystagmus, an involuntary rhythmic movement of the eyes, was observed in several patients and represents an important clinical finding that can significantly impact functional vision even when visual acuity measurements suggest reasonable remaining vision. Nystagmus in RP patients may result from the loss of stable fixation due to central retinal involvement, or it may represent a congenital condition that coexists with RP, particularly in cases of early-onset disease.

Regarding systemic associations, several patients reported conditions that may indicate syndromic forms of RP. Hearing loss was documented in multiple patients, raising the possibility of Usher syndrome, which represents one of the most common syndromic forms of RP and is characterized by the combination of RP with sensorineural hearing loss. The identification of *USH2A* mutations in some patients supports this possibility, as this gene is a major cause of Usher syndrome type 2. The presence of hearing loss in RP patients has significant implications for diagnosis, genetic counseling, and comprehensive care planning, as it may indicate specific genetic subtypes with different inheritance patterns and prognosis.

Other systemic conditions reported included diabetes mellitus and hypertension, which are common in the general population but may have particular relevance for RP patients due to potential effects on retinal vascular health and overall disease progression. While these conditions are likely coincidental in most cases, their presence requires careful management to avoid additional retinal damage from diabetic retinopathy or hypertensive retinopathy that could compound the vision loss from RP.

## 4. Discussion

### 4.1. Epidemiological Characteristics and Population-Specific Patterns

The epidemiological findings from this Turkish RP cohort reveal distinctive characteristics that reflect both the genetic architecture of inherited retinal dystrophies and the cultural and demographic factors unique to this cohort of 95 Turkish RP patients. The mean symptom onset age of 14.8 years represents a significantly earlier disease manifestation compared to many Western populations, where mixed inheritance patterns typically result in later average onset ages. This early onset pattern strongly suggests a predominance of autosomal recessive inheritance forms, which characteristically manifest during childhood and adolescence rather than adulthood [15,20].

The early onset finding has profound implications for healthcare systems, educational institutions, and social support structures in Turkey. When a substantial proportion of RP patients experience their first symptoms during school-age years, this creates unique challenges for educational planning, career counseling, and psychological support during critical developmental periods. The identification of visual impairment during childhood also places significant stress on families, who must navigate both the emotional impact of a progressive vision loss diagnosis and the practical challenges of ensuring appropriate educational and social development for their affected children.

The high rate of positive family history (53.1%) observed in this study exceeds typical rates reported from populations with lower consanguinity levels, providing strong evidence for the role of genetic factors in shaping RP epidemiology in Turkey. This finding suggests that genetic counseling needs in Turkey may be particularly acute, as many families will have multiple affected members and therefore face repeated decisions about family planning, genetic testing, and risk assessment for future children. The clustering of cases within families also creates opportunities for more efficient genetic testing strategies, where identification of causative mutations in one family member can guide testing and counseling for other relatives.

The geographical and demographic diversity of the patient population, while not formally analyzed in this study, likely reflects the complex population structure of Turkey, which includes both urban populations with more diverse genetic backgrounds and rural populations that may have experienced greater genetic isolation and founder effects. Understanding these population substructures may be important for developing targeted prevention and treatment strategies that account for regional variations in disease prevalence, genetic causes, and cultural factors affecting healthcare utilization.

### 4.2. The Profound Impact of Consanguineous Marriages on RP Epidemiology

The 52.4% rate of consanguineous marriages among RP patients represents one of the most striking findings of this study and provides compelling evidence for the causal relationship between consanguinity and inherited retinal dystrophy risk. This rate is approximately 2.8 times higher than the general Turkish population average, representing a substantial elevation that cannot be explained by chance alone [13]. The magnitude of this association suggests that consanguineous marriage may be one of the most important modifiable risk factors for this cohort of 95 Turkish patients with RP.

The mechanisms underlying this association are well-established in population genetics theory. Consanguineous marriages increase the probability that offspring will inherit identical copies of recessive alleles from both parents, who share these alleles through common ancestry [13,14]. For autosomal recessive conditions like many forms of RP, this dramatically increases the risk of disease expression compared to non-consanguineous unions. The specific types of consanguineous relationships observed in this study, particularly first cousin marriages, result in offspring sharing approximately 12.5% of their genome through common ancestry, creating substantial risk elevations for recessive genetic conditions.

The cultural and historical context of consanguineous marriage in Turkey reflects complex social, economic, and religious factors that have maintained these practices across generations despite increasing urbanization and modernization. Traditional preferences for cousin marriage have been reinforced by practical considerations such as property consolidation, family unity maintenance, and cultural continuity preservation. However, the health consequences of these practices, as demonstrated by the elevated RP rates observed in this study, suggest an urgent need for culturally sensitive educational interventions and genetic counseling programs.

The implications of these findings extend far beyond individual families to encompass public health policy, healthcare resource allocation, and prevention strategy development. The nearly threefold increase in RP risk associated with consanguineous marriage suggests that targeted interventions focused on high-consanguinity communities could have substantial impact on disease prevention. Such interventions might include pre-marital genetic screening programs, carrier testing initiatives, and comprehensive genetic counseling services that help couples make informed decisions about family planning while respecting cultural values and preferences.

The success of prevention programs will likely depend on the development of culturally appropriate educational materials and counseling approaches that acknowledge the complex motivations for consanguineous marriage while providing clear, accurate information about genetic risks. Collaboration with religious leaders, community elders, and cultural organizations may be essential for developing effective prevention strategies that are acceptable within traditional Turkish communities while promoting informed decision-making about marriage and reproduction.

### 4.3. Disease Severity Patterns and Clinical Progression Characteristics

The predominance of severe vision loss (85.7%) among patients with available visual acuity data represents a particularly concerning finding that has multiple important implications for healthcare planning, resource allocation, and patient care strategies. This severity distribution suggests that this cohort of 95 Turkish patients with RP may be characterized by either more rapid progression rates, earlier onset of severe complications, or ascertainment bias toward more severely affected patients who are more likely to seek specialized care or participate in research studies.

Several factors may contribute to the observed severity pattern in this Turkish cohort. The predominance of autosomal recessive inheritance forms, which typically progress more rapidly and severely than autosomal dominant variants, likely plays a significant role in the advanced disease stage observed in many patients [15,21]. Autosomal recessive RP often manifests with earlier onset, more extensive retinal involvement, and faster progression to legal blindness compared to dominant forms, which may explain the high proportion of severely affected patients in this study.

The potential role of specific genetic variants or founder mutations in contributing to severe phenotypes deserves particular consideration. Populations with elevated consanguinity rates and genetic isolation may experience the increased prevalence of specific mutations that are associated with particularly severe clinical courses [28,29]. If certain mutations common in Turkey result in rapid progression or early-onset severe disease, this could explain the observed severity distribution. Comprehensive genetic analysis of this population, including genotype–phenotype correlation studies, would be valuable for understanding these relationships.

While genetics and high consanguinity likely contribute to severe vision loss, alternative explanations such as delayed diagnosis, limited access to early intervention, and selection bias toward severe phenotypes in specialty clinics must also be considered.

The advanced disease stage observed in many patients also raises important questions about healthcare access, early diagnosis capabilities, and intervention timing in Turkey. Late diagnosis due to limited awareness of RP symptoms, inadequate access to specialized ophthalmological care, or delayed recognition of hereditary vision loss could contribute to the presentation of patients with advanced disease [30]. Alternatively, the severity pattern might reflect the natural history of RP in this population, suggesting that disease progression is indeed more rapid or severe than in other populations due to genetic factors.

The clinical implications of these severity findings are substantial for healthcare system planning and patient care strategies. The high proportion of patients with severe vision loss indicates significant needs for low-vision rehabilitation services, orientation and mobility training, assistive technology provision, and psychosocial support services [17,19]. Educational systems must be prepared to accommodate students with significant visual impairments, and employment support programs may be needed to help affected individuals maintain productive careers despite progressive vision loss.

### 4.4. Genetic Architecture and Molecular Diagnostic Insights

The genetic testing results provide valuable insights into both the molecular landscape of RP in Turkey and the evolving capabilities of genetic diagnostics in Turkish healthcare. The 54.3% rate of genetic testing among patients with detailed data represents a relatively high utilization rate that likely reflects increasing awareness of genetic testing importance, improving access to molecular diagnostic services, and growing recognition of genetic diagnosis value for patient counseling and family planning [22,23].

The specific genes identified in this Turkish cohort reveal interesting patterns that may reflect population-specific genetic architecture. The prominence of *CERKL* and *USH2A* as identified causes aligns with theoretical expectations for populations with elevated consanguinity [24,26], where founder effects and genetic drift can lead to increased frequencies of specific mutations. *CERKL* mutations have been previously reported as significant causes of autosomal recessive RP in Middle Eastern populations, while *USH2A* represents one of the most common causes of Usher syndrome worldwide, suggesting that this gene may be particularly important in the Turkish RP population.

The identification of *MERTK* mutations in Turkish patients aligns with previous reports suggesting this gene as a significant cause of RP in Turkish and other Middle Eastern populations [24,25]. *MERTK* encodes a receptor tyrosine kinase involved in photoreceptor outer segment phagocytosis, and mutations in this gene typically result in autosomal recessive RP with characteristic clinical features. The presence of this gene among identified causes supports the importance of including population-specific gene panels in diagnostic testing strategies for Turkish patients.

The diversity of identified genes, including *CRB1*, *ABCA4*, and others, reflects the substantial genetic heterogeneity that characterizes RP in all populations studied to date. However, the specific frequency distribution of these genes may indicate population-specific patterns that could inform targeted genetic testing strategies. The development of gene panels optimized for Turkish patients, based on the frequency patterns observed in this and other Turkish studies, could improve diagnostic efficiency and cost-effectiveness while ensuring comprehensive coverage of relevant genetic variants.

The molecular diagnostic findings also highlight the importance of comprehensive genetic testing approaches, including both targeted gene sequencing and broader genomic analysis methods such as whole exome or whole genome sequencing. As genetic testing technologies continue to evolve and costs decrease, more comprehensive approaches may become standard practice, potentially identifying rare variants or novel genes that contribute to RP in specific populations like Turkey.

### 4.5. Comparative Analysis with International Populations and Phenotypic Variations

Recent investigations into the phenotypic variability of retinal disease among populations with genetic variants in related genes have revealed important insights into the spectrum of clinical manifestations and disease severity patterns observed across different patient cohorts [29]. The findings from our Turkish population complement international research demonstrating that genetic background and population-specific factors significantly influence disease expression and progression patterns. The divergent manifestations observed in biallelic versus monoallelic variants of RP-associated genes underscore the complex genetic architecture underlying inherited retinal disorders [31].

Comparative analysis of our Turkish cohort with data from other populations suggests that the particularly aggressive disease phenotype observed in our patients may reflect the influence of specific founder mutations or the cumulative effect of autosomal recessive inheritance patterns prevalent in this population. The natural history of DHDDS-associated RP59 and other rare genetic subtypes identified through recent comprehensive genetic studies provides a framework for understanding disease progression patterns and planning long-term clinical management strategies [32].

The clinical presentations of syndromic forms of RP, particularly those associated with systemic manifestations such as X-linked CGD chorioretinitis observed in affected individuals, demonstrate the importance of considering systemic factors when evaluating inherited retinal dystrophies [33]. The comprehensive evaluation of our Turkish cohort similarly identified multiple patients with syndromic associations, including Usher syndrome, highlighting the necessity of multidisciplinary assessment approaches and consideration of extraocular manifestations in RP diagnosis and management.

### 4.6. Therapeutic Implications and Future Treatment Considerations

The genetic landscape revealed by this study has important implications for current and emerging therapeutic approaches for RP. The identification of specific genes as significant causes of this cohort of 95 Turkish patients with RP shows provides targets for gene-specific therapeutic development and indicates which patients might benefit from existing or developing treatments. The prominence of genes such as *USH2A* and *CERKL* suggests that therapeutic approaches targeting these specific molecular pathways could have substantial impact on the Turkish RP population.

Recent advances in therapeutic approaches, including CRISPR gene editing technologies that have demonstrated efficacy in preclinical models of retinitis pigmentosa, offer promising avenues for population-specific therapeutic development [34,35]. The potential application of anti-anemia drug targets in RP treatment, as demonstrated through independent preclinical models, suggests novel therapeutic strategies that may be particularly relevant for autosomal recessive forms prevalent in our Turkish cohort [34].

Current FDA-approved gene therapy approaches, such as the RPE65-targeted therapy approved for Leber congenital amaurosis, may have limited applicability to this Turkish cohort based on the genetic spectrum observed [36,37]. However, the identification of specific genes prevalent in this population could guide the development of targeted therapeutic approaches or indicate Turkish patients who might benefit from emerging gene therapies as they become available. The relatively high rate of genetic testing also suggests that Turkish patients are increasingly likely to have molecular diagnoses that could guide treatment decisions.

Emerging therapeutic approaches beyond gene therapy, including investigation of novel molecular targets such as miR-181a/b in retinitis pigmentosa with implications for disease progression and therapy development, may offer additional treatment options for our patient population [38]. The modulation of RPE glycolysis through VHL manipulation in rod photoreceptors represents another promising preclinical approach that has demonstrated efficacy in improving disease phenotypes in experimental RP models [39].

The importance of comprehensive clinical trial participation and therapeutic research involving Turkish patients cannot be overstated. Population-specific genetic variants, environmental factors, and cultural considerations may influence therapeutic responses in ways that are not apparent from studies conducted in other populations. Encouraging participation in clinical trials and ensuring that Turkish patients have access to experimental therapies through appropriate research protocols will be important for advancing treatment options for this population.

### 4.7. Prevention Strategies and Public Health Interventions

The strong association between consanguineous marriage and RP risk identified in this study suggests that prevention-focused interventions could have substantial impact on reducing disease incidence in Turkey. However, the development of effective prevention strategies requires careful consideration of cultural sensitivities, religious considerations, and the complex social factors that maintain traditional marriage practices across generations.

Pre-marital genetic screening represents one potential prevention strategy that could be particularly effective in the Turkish context, where arranged marriages and family involvement in marriage decisions remain common in many communities. Systematic carrier testing for common RP-causing mutations, particularly those identified as frequent in Turkish populations, could provide couples with information needed to make informed decisions about marriage and reproduction. The success of such programs would depend on the development of culturally appropriate counseling approaches and the integration of genetic testing into existing pre-marital health assessment practices.

Educational interventions targeting healthcare providers, religious leaders, and community organizations could help increase awareness of genetic risks associated with consanguineous marriage while respecting cultural values and traditions. Such programs should emphasize that genetic counseling and testing provide information to support informed decision-making rather than prohibiting traditional practices and should acknowledge the complex motivations for cousin marriage that extend beyond simple tradition to include practical considerations such as family unity and economic factors.

The development of comprehensive genetic counseling services specifically designed for Turkish cultural contexts represents another important prevention opportunity. Counseling approaches that incorporate religious perspectives, acknowledge family decision-making structures, and provide culturally appropriate risk communication strategies may be more effective than generic counseling protocols developed for other populations. Training programs for genetic counselors working with Turkish populations should emphasize cultural competency and sensitivity to traditional values while ensuring accurate communication of genetic risk information.

Population-based screening and surveillance programs could also contribute to prevention efforts by identifying at-risk individuals before symptom onset, enabling early intervention and family counseling. School-based vision screening programs enhanced with RP-specific protocols could potentially identify affected children earlier in the disease course, facilitating prompt medical care and family genetic counseling. Such programs would need to balance the benefits of early detection against the potential psychological and social consequences of diagnosing progressive vision loss in children.

### 4.8. Study Limitations and Methodological Considerations

Several important limitations must be acknowledged when interpreting the findings from this study. The retrospective design introduces potential recall bias, especially for variables reliant on patient or family recollection. Sample size limitations restrict generalizability for statistical analyses. Selection bias represents another key limitation, as patients were recruited through specialized ophthalmology clinics and patient support organizations, which may enrich the sample for more severely affected individuals who actively seek medical care or participate in patient advocacy activities. Further, as patients were recruited through specialized ophthalmology clinics and patient support organizations, milder cases and those without access to specialized care may be underrepresented. This may result in overestimation of disease severity and rates of consanguinity for the broader Turkish RP population. Patients with milder diseases who have not yet sought specialized care, who are unaware of their diagnosis, or who do not participate in patient organizations may be underrepresented in this cohort, potentially affecting the generalizability of findings regarding disease severity and progression patterns. Genetic testing coverage and regional representation were also incomplete, limiting broader conclusions for the Turkish RP population.

Sample size limitations, particularly for certain analyses such as visual acuity assessment, restrict the generalizability of some findings and limit statistical power for detecting associations or differences between subgroups. Visual acuity measurements were available in only a subset *(n* = 21), which may limit generalizability of findings regarding the distribution and severity of vision loss. The limited dataset may introduce bias if patients with more severe disease are more likely to have recent ophthalmological evaluations and documented visual acuity, and consequently, our pattern of vision loss observed may not be fully representative of the broader Turkish RP population.

Selection bias represents another important limitation, as patients were recruited through specialized ophthalmology clinics and patient support organizations, which may enrich the sample for more severely affected individuals who actively seek medical care or participate in patient advocacy activities. Patients with milder diseases who have not yet sought specialized care, who are unaware of their diagnosis, or who do not participate in patient organizations may be underrepresented in this cohort, potentially affecting the generalizability of findings regarding disease severity and progression patterns.

Out of 95 patients in the cohort, only 52 (54.7%) underwent genetic testing, and molecular diagnoses were available only for some; therefore, genetic findings should be interpreted with caution. The patients who underwent genetic testing may not be representative of the entire cohort, as testing decisions are influenced by factors such as healthcare access, insurance coverage, physician recommendations, and patient preferences.

Regional representation within Turkey may be incomplete, as patient recruitment was not systematically designed to ensure proportional representation from all geographical regions or demographic subgroups. Regional representation was incomplete and not systematically stratified, and thus, findings may not reflect prevalence or patterns in underrepresented provinces or communities. Rural populations, ethnic minorities, and individuals from lower socioeconomic backgrounds may be underrepresented, potentially affecting the generalizability of findings regarding consanguinity patterns, disease severity, and healthcare utilization patterns. Future studies should implement systematic sampling strategies to ensure broader population representation.

The lack of standardized clinical assessments, including detailed ophthalmological examinations, electroretinography, visual field testing, and other objective measures of retinal function, represents a significant limitation that restricts the ability to characterize disease severity and progression patterns accurately. The absence of standardized clinical assessments and reliance, in some cases, on patient-reported visual acuity introduces variability and potential bias to severity estimates.

### 4.9. Future Research Priorities and Directions

Based on the findings and limitations of this study, several important research priorities emerge that could significantly advance understanding of RP in Turkey and improve clinical care for affected patients. Prospective longitudinal studies designed to track disease progression over time represent perhaps the most critical research need, as understanding natural history patterns is essential for patient counseling, treatment planning, and clinical trial design. Such studies should incorporate standardized clinical assessments, objective measures of retinal function, and systematic genetic analysis to provide comprehensive characterizations of disease progression patterns.

Comprehensive genetic analysis of larger patient cohorts using advanced sequencing technologies represents another high priority research direction. Whole exome or whole genome sequencing studies could provide definitive characterizations of the genetic landscape of RP in Turkey, identify population-specific variants, and potentially discover novel genes or mutations that contribute to disease in this population [23]. Such studies should include both affected individuals and unaffected family members to enable comprehensive segregation analysis and variant interpretation.

Genotype–phenotype correlation studies linking specific genetic variants to clinical presentation patterns, disease severity, and progression rates could provide valuable insights for patient counseling and treatment planning. Understanding whether specific mutations common in Turkey are associated with more rapid progression, earlier onset, or particular clinical features could inform genetic counseling protocols and help establish prognosis for newly diagnosed patients. Such studies require large, well-characterized patient cohorts with both comprehensive genetic analysis and detailed longitudinal clinical assessment.

Future prospective studies should aim to perform detailed genotype–phenotype correlation to elucidate the relationships between genetic variants and clinical presentation.

Multi-center collaborative studies involving multiple regions of Turkey could address the regional representation limitations of this study while providing sufficient sample sizes for robust statistical analysis of factors affecting disease presentation and progression. Collaboration with international research consortiums could also provide opportunities for comparative analyses that illuminate population-specific factors affecting RP epidemiology and clinical presentation.

Population-based epidemiological studies designed to establish accurate prevalence estimates for RP and other inherited retinal dystrophies in Turkey would provide essential information for healthcare planning and resource allocation. Such studies should use systematic sampling methodologies to ensure representative population coverage and should incorporate both clinical assessment and genetic analysis components to provide comprehensive characterizations of disease burden.

Intervention studies evaluating the effectiveness of genetic counseling programs, prevention strategies, and clinical care protocols specifically designed for Turkish populations represent important applied research priorities. Understanding which approaches are most effective for increasing genetic testing utilization, improving family planning decision-making, and enhancing clinical outcomes could guide the development of evidence-based care protocols optimized for Turkish patients and families.

## 5. Conclusions

This comprehensive analysis of 95 RP patients represents the first systematic characterization of inherited retinal dystrophy patterns in this cohort of 95 Turkish RP patients and reveals several distinctive features that have important implications for clinical care, public health policy, and research priorities. The study findings provide compelling evidence for population-specific factors that significantly influence RP epidemiology, clinical presentation, and healthcare needs in Turkey.

The early symptom onset pattern, with a mean age of 14.8 years and 59.5% of patients experiencing first symptoms before age 15, highlights the pediatric nature of RP in Turkey and underscores the critical importance of childhood awareness, early diagnostic capabilities, and family-centered care approaches. This pattern strongly suggests a predominance of autosomal recessive inheritance forms, which characteristically manifest during childhood and adolescence rather than adulthood, fundamentally altering the clinical approach needed for Turkish patients compared to populations where later-onset dominant forms are more prevalent [2,15].

The extraordinarily high rate of positive family history (53.1%) and consanguineous marriage (52.4%) provide compelling evidence for the genetic architecture underlying RP in Turkey and demonstrates the profound impact of cultural practices on disease epidemiology [10,11,12,29]. The nearly threefold elevation in consanguinity rates among RP patients compared to the general Turkish population represents one of the strongest associations between cultural practices and genetic disease risk documented in any population, highlighting both the challenges and opportunities for prevention-focused interventions [8,13].

The predominance of severe vision loss (85.7% of patients with visual acuity data) indicates that RP in Turkey may be characterized by particularly aggressive disease progression or that healthcare systems must be prepared to address substantial needs for comprehensive rehabilitation services, assistive technologies, and psychosocial support. This severity pattern has immediate implications for resource allocation, service planning, and the development of specialized care capabilities tailored to the needs of patients with advanced visual impairment [17,19].

The genetic testing results, while preliminary due to limited sample size, provide valuable insights into the molecular landscape of RP in Turkey and suggest that population-specific genetic variants may play important roles in disease causation. The identification of genes such as *CERKL*, *USH2A*, and *MERTK* as significant causes in this population provides targets for therapeutic development and indicates which patients might benefit from emerging gene-specific treatments as they become available [23,28].

These findings collectively demonstrate that RP in Turkey exhibits distinctive epidemiological and clinical characteristics that require population-specific approaches to prevention, diagnosis, treatment, and care. The high rates of consanguinity and early symptom onset create both challenges and opportunities for intervention, suggesting that culturally sensitive prevention programs could have substantial impact on reducing disease incidence while comprehensive pediatric care capabilities are essential for managing affected children and their families.

The implications of these findings extend far beyond clinical medicine to encompass public health policy, educational system planning, social services development, and research prioritization. Healthcare systems must be prepared to address the complex needs of patients with early-onset progressive vision loss, including specialized pediatric services, comprehensive genetic counseling programs, and extensive rehabilitation services. Educational institutions require enhanced capabilities for accommodating students with progressive visual impairment, while social support systems must address the unique challenges faced by families dealing with hereditary vision loss.

Looking toward the future, these findings provide a foundation for developing evidence-based approaches to RP prevention, diagnosis, and treatment that are specifically tailored to Turkish population needs and characteristics. The success of such approaches will depend on continued research efforts, healthcare system development, and the implementation of culturally sensitive interventions that respect traditional values while promoting informed decision-making about genetic risks and family planning.

Understanding population-specific characteristics of inherited retinal dystrophies represents a critical component of personalized medicine approaches and precision public health strategies. The distinctive patterns observed in Turkish RP patients demonstrate the importance of population-based research for developing effective prevention and treatment strategies, and these findings contribute valuable insights to the global understanding of inherited retinal dystrophy epidemiology and management. As genetic therapies and other advanced treatments continue to evolve, having detailed knowledge of population-specific disease patterns will become increasingly important for ensuring that all patients have access to appropriate, effective care tailored to their specific genetic and cultural contexts.

## Figures and Tables

**Table 1:** Genes Identified in RP Cohort and Frequencies.

Gene	Mutation Type	Inheritance Pattern	N	Percentage (%)	Clinical Relevance/Notes
*CERKL*	Loss-of-function	Autosomal recessive	2	10.5 *	Common AR RP/CRD gene
*USH2A*	Missense/LOF	Autosomal recessive	2	10.5 *	Usher syndrome type 2/non-syndromic
*MERTK*	Loss-of-function	Autosomal recessive	1	5.3 *	Severe early-onset RP
*CRB1*	Missense/LOF	Autosomal recessive	1	5.3 *	RP/Leber congenital amaurosis
*ABCA4*	Missense/LOF	Autosomal recessive	1	5.3 *	RP/Stargardt-like phenotype

* Percentages are calculated from the subset of genetically tested patients (*n* = 19). Table 1 summarizes all genes and their variants identified among genetically tested RP patients in this cohort. *CERKL* and *USH2A* were the most commonly found mutations, followed by single cases involving *MERTK, CRB1*, and *ABCA4.* Percentages reflect only the subset who underwent genetic testing. Data to be referenced from the Section 3 where genetic findings are reported.

## Data Availability

The original contributions presented in this study are included in the article. Further inquiries can be directed to the corresponding author.

## Abbreviations

RP	Retinitis Pigmentosa
SD	Standard Deviation
IRB	Institutional Review Board
ERG	Electroretinography
VA	Visual Acuity
AR	Autosomal Recessive
AD	Autosomal Dominant
XL	X-linked
DNA	Deoxyribonucleic Acid
PCR	Polymerase Chain Reaction
*CERKL*	Ceramide Kinase Like (gene)
*USH2A*	Usher Syndrome Type 2A (gene)
*CRB1*	Crumbs Cell Polarity Complex Component 1 (gene)
*ABCA4*	ATP Binding Cassette Subfamily A Member 4 (gene)
*MERTK*	MER Proto-Oncogene, Tyrosine Kinase (gene)
